# Evaluation of an educational video assessing knowledge and attitudes of transgender youth on fertility preservation

**DOI:** 10.1093/pch/pxae050

**Published:** 2025-03-24

**Authors:** Florence Grégoire-Briard, Andie Chernoff, Ken Tang, Jenna Gale, Margaret L Lawson, Maria Kielly

**Affiliations:** Gynecology Department, Hôpital du Sacré-Coeur de Montréal, Montréal, Québec, Canada; Department of Obstetrics and Gynecology, University of Montréal, Montréal, Québec, Canada; Department of Medicine, University of Ottawa, Ottawa, Canada; Independent statistical consultant, Richmond, British Columbia, Canada; Department of Obstetrics and Gynecology, University of Ottawa, Ottawa, Canada; Ottawa Fertility Centre, Ottawa, Canada; CHEO Research Institute, CHEO (Children’s Hospital of Eastern Ontario), Ottawa, Canada; Department of Obstetrics and Gynecology, Memorial University, Saint-Johns’, Newfoundland and Labrador, Canada

**Keywords:** *Educational video*, *Fertility preservation*, *Transgender care*

## Abstract

**Objectives:**

While gender-affirming therapy can alleviate gender dysphoria in transgender individuals, such treatments may negatively impact fertility. As such, multiple organizations have highlighted the importance of counselling and offering fertility preservation (FP) prior to the initiation of medical therapy. To supplement fertility discussions, whiteboard educational videos were created to review and provide information on FP for transgender and non-binary youths and their families. This study assessed the understandability, actionability, and readability of the FP educational videos; patient perceptions, knowledge, barriers, and interest in FP; and overall satisfaction with the videos.

**Methods:**

Participants (age 12 to 18) completed an online survey assessing their knowledge, perceptions, and overall thoughts on FP prior to watching a short educational video on FP. They were then invited to complete the survey again after watching the educational video and provide feedback on the video presented.

**Results:**

Twenty-one participants were enrolled in the study. Seventeen participants completed the pre-video survey, and 12 completed the post-video and video feedback survey (completion rate of 81.0% and 57.1%, respectively). The mean age of participants was 15.50 (SD = 1.34). In the pre-video survey, one participant (1/15; 6.7%) rated their knowledge of FP as ‘very good’ or ‘good’ (1/15; 6.7%) compared to 10 participants (10/12; 83.3%) in the post-video survey (P = 0.039). Feedback regarding the videos was overwhelmingly positive.

**Conclusions:**

Our results suggest that targeted patient education aids, such as a whiteboard educational video on FP, can be used to supplement fertility discussions with gender-diverse youth.

Hormone therapy and surgery may be used to help alleviate gender dysphoria and allow transgender individuals to achieve physical changes consistent with their affirmed gender (1). However, such treatments can negatively impact spermatogenesis and oocyte production; thus, the Endocrine Society, World Professional Association for Transgender Health, and American Society for Reproductive Medicine guidelines emphasize the importance of counselling about infertility risk and offering fertility preservation (FP) prior to starting gender-affirming therapy (2–4). These conversations may be challenging at any age, but particularly amongst our pediatric and adolescent gender-diverse population. Members of the Gender Diversity Program at our institution, a tertiary care pediatric centre in an urban setting, created short whiteboard videos to explain the relevant anatomy, physiology of fertility, and process of cryopreservation and FP to transgender youths and their families in order to facilitate those discussions. This study aimed to (1) assess the understandability, actionability, and readability of the video; (2) assess patient perceptions, knowledge, barriers, and interest about FP; and (3) evaluate overall patient satisfaction with the educational videos.

## METHODS

Institutional Review Board (IRB) approval was obtained. Patients (age 12 to 18 years at enrollment) presenting for either their first consultation or follow-up at our institution’s Gender Diversity clinic from December 1, 2021, to June 1, 2023, were approached for participation in the study. Patients were required to be fluent in English or in French. Sex assigned at birth and current gender identity were irrelevant to the recruitment of participants (transmale, transfemale, non-binary, queer, and gender fluid patients were included in the recruitment process). Participants had to have the ability to consent in order to participate in the study. Allied healthcare members of our gender diversity team (social worker, nurse, pediatric endocrinologist, pediatric gynecologist) were asked to participate in the recruitment of patients for this study. Allied providers were given general information on the study and asked to address the study with all their patients coming for new consultations and follow-ups (either in person or virtually, at the discretion of the treating provider) during the study period. With patient consent/interest, contact information was then relayed to the study team. Records of those discussions and data of the number of patients approached in the clinic were not collected.

Study participants were emailed a link to a short pre-video survey assessing their demographics and a general questionnaire on FP. The questionnaire was developed by our team, as there are no validated tools specific to this topic, to assess patient perceptions, knowledge, and interest in FP. Following completion of the pre-video survey, they were invited to watch a short whiteboard educational video on FP, in either English or French, corresponding to their assigned sex at birth. Finally, they were asked to complete a post-video survey assessing knowledge and interest in FP after watching the video, as well as give their overall impression and feedback on the video.

**Figure 1.**
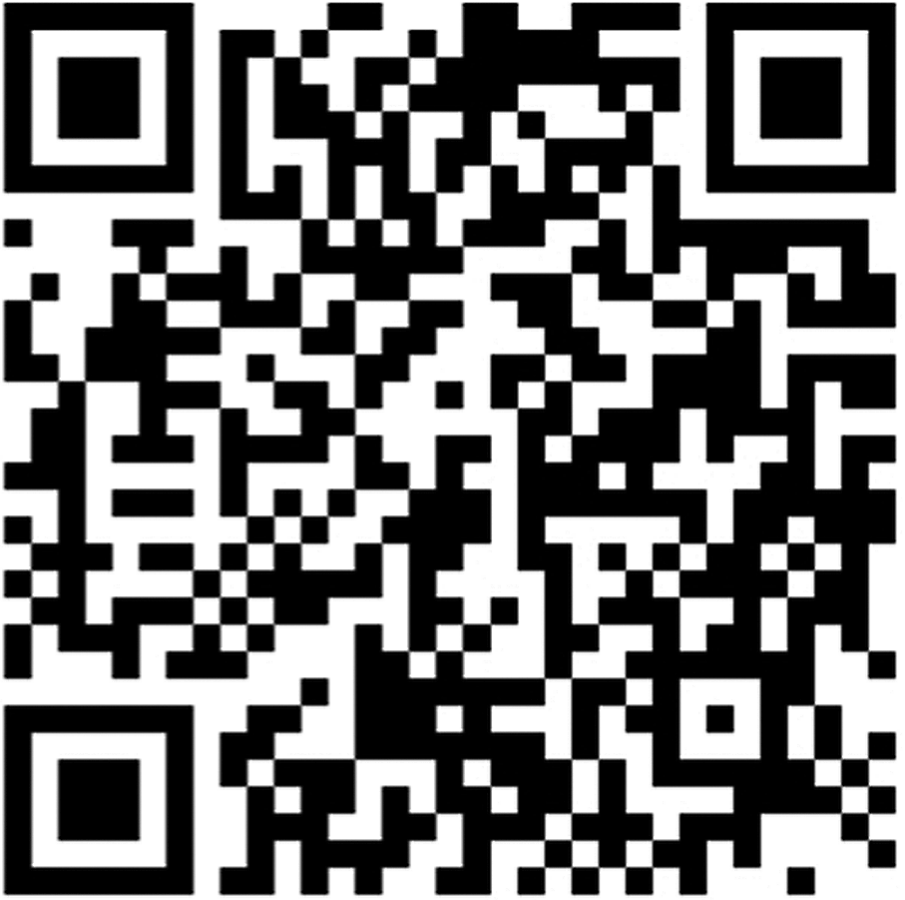
QR code to access FP video for assigned female at-birth patients

**Figure 2.**
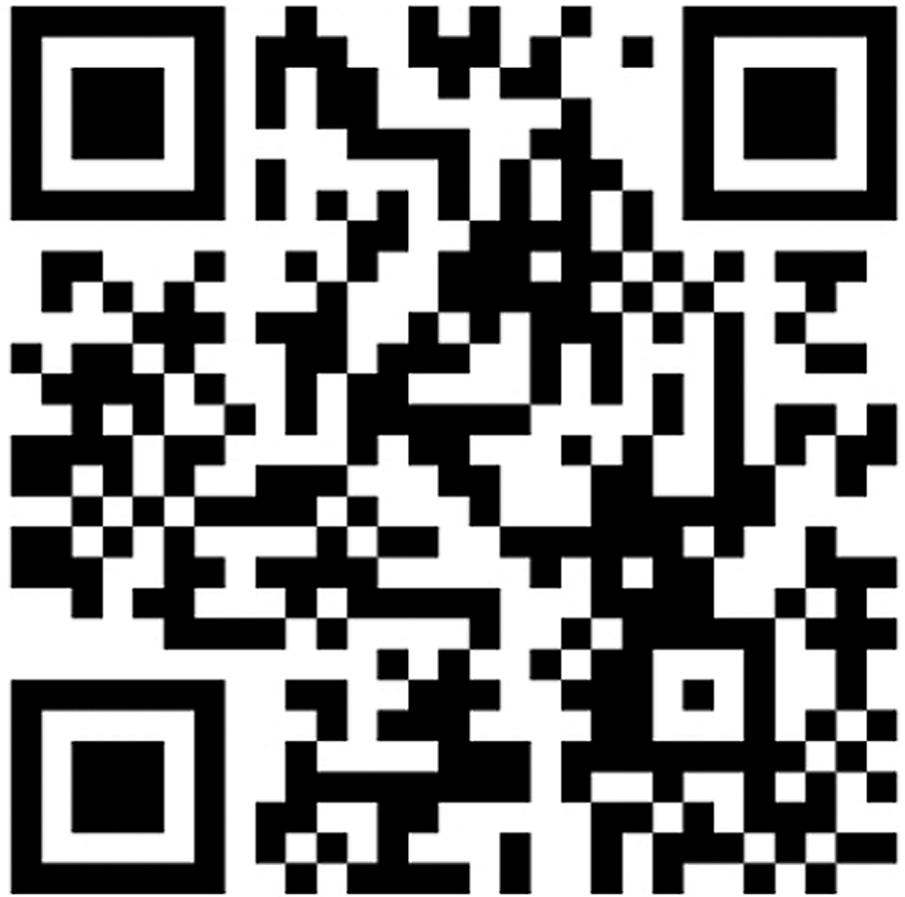
QR code to access FP video for assigned male at-birth patients

Video scripts were developed by two pediatric and adolescent gynecologists, a consulting reproductive and fertility specialist involved with FP, and a pediatric endocrinologist. Scripts were based on clinical experience with patients and major concepts in the transgender literature. The scripts (one for assigned female at birth and one for assigned male at birth) were reviewed by a provincial trans advocacy group to assist with content and language. QR codes to access the educational videos are provided in Figures 1 and 2.

## RESULTS

Twenty-one participants (21) were enrolled in the study. The mean age of participants was 15.5 (SD = 1.3). Fifteen participants (15/16; 93.8%) listed Canada as their country of birth. The majority of participants (13/16; 81.2%), were in high school, but two (2/16; 12.5%) were in elementary school, and one (1/16; 6.3%) reported ‘other’ for their education level. Seven participants identified as ‘trans male’ (7/17; 41.2%), eight identified as ‘trans female’ (8/17; 47.1%), and two identified as ‘other’ (2/17; 11.8%).

### Pre-video survey

Seventeen participants (17/21; completion rate of 81.0%) attempted the pre-video survey questions (i.e., responded to at least one of the pre-video items). FP knowledge was rated as ‘very good’ or ‘good’ by one participant (1/15; 6.7%). Six participants (6/15; 40.0%) were ‘very interested’ or ‘interested’ in knowing more about FP. Regarding the importance of having their own biological children, one participant (1/15; 6.7%) endorsed the importance. When asked about discussing FP with a health care provider, only five participants (5/15; 33.3%) reported feeling ‘comfortable’ or ‘very comfortable’ addressing the issue. Only two participants (2/15; 13.3%) were ‘interested’ or ‘very interested’ in pursuing FP. Results between the Assigned Female at Birth (AFAB) group and the Assigned Male at birth (AMAB) groups were similar, as demonstrated in Table 1, although the sample size were too small in each category to draw formal conclusions. Barriers to pursuing FP included: not something the participant would need (11/17; 64.7%), interest in adoption (8/17; 47.1%), potential delays in hormonal therapy (8/17; 47.1%), and feeling uncomfortable with the procedure (7/17; 41.2%) (participants were allowed to report more than one barrier if applicable).

**Table 1.** Pre-video survey results

Variable	Total population		AFAB		AMAB	
	n	Freq (%)	n	Freq (%)	n	Freq (%)
How would you rate your knowledge of fertility preservation (FP)?	15		7		8	
Very good		1 (6.7)		1 (14.3)		0 (0.0)
Good		0 (0.0)		0 (0.0)		0 (0.0)
Fair		10 (66.7)		6 (85.7)		4 (50.0)
Not very good		4 (26.7)		0 (0.0)		4 (50.0)
Not at all		0 (0.0)		0 (0.0)		0 (0.0)
How would you describe your knowledge of FP?	**15**		7		8	
I know and understand the specific details of FP		1 (6.7)		1 (14.3)		0 (0.0)
I know general principles but missing specific details		6 (40.0)		5 (71.4)		1 (12.5)
I have limited knowledge of FP		7 (46.7)		1 (14.3)		6 (75.0)
No knowledge of FP		1 (6.7)		0 (0.0)		1 (12.5)
How interested are you in knowing more about fertility preservation?	**15**		7		8	
Very interested		1 (6.7)		0 (0.0)		1 (12.5)
Interested		5 (33.3)		3		2 (25.0)
Neutral		3 (20.0)		0 (0.0)		3 (37.5)
Not interested		3 (20.0)		2 (28.6)		1 (12.5)
Not at all		3 (20.0)		2 (28.6)		1 (12.5)
How important is it for you to have your own biological child?	**15**		7		8	
Very important		0 (0.0)		0 (0.0)		0 (0.0)
Important		1 (6.7)		0 (0.0)		1 (12.5)
Neutral		0 (0.0)		0 (0.0)		0 (0.0)
Not important		14 (93.3)		7 (100.0)		7 (87.5)
I’m not sure		0 (0.0)		0 (0.0)		0 (0.0)
How comfortable are you discussing FP with a healthcare provider	**15**		7		8	
Very comfortable		2 (13.3)		1 (14.3)		1 (12.5)
Comfortable		3 (20.0)		1 (14.3)		2 (25.0)
Neutral		8 (53.3)		3 (42.9)		5 (62.5)
Not Comfortable		2 (13.3)		2 (28.6)		0 (0.0)
Not at all		0 (0.0)		0 (0.0)		0 (0.0)
Are you interested in pursuing FP treatment?	15		7		8	
Very interested		1 (6.7)		0 (0.0)		1 (12.5)
Interested		1 (6.7)		1 (14.3)		0 (0.0)
Neutral		1 (6.7)		0 (0.0)		1 (12.5)
Not interested		5 (33.3)		1 (14.3)		4 (50.0)
Not at all		7 (46.7)		5 (71.4)		2 (25.0)

### Post-video survey

Twelve participants (12/21; completion rate of 57.1%) attempted (responded to at least 1 item) in the post-video survey. FP knowledge post-video was rated as ‘very good’ or ‘good’ by 10 participants (10/12; 83.3%), which represented a statistically significant difference from the pre-video survey (P = 0.039) in which only 6.7% of participants rated their knowledge of FP as ‘very good’ or ‘good’. Two participants (2/11; 18.2%) were interested in learning more about FP after watching the video and seven participants (7/12; 58.3%) felt comfortable addressing this topic with their healthcare provider. Results between the AFAB group and the AMAB group were similar, as demonstrated in Table 2, although the sample size was too small in each category to draw formal conclusions. Finally, one participant (1/12; 8.33%) reported being ‘very interested’ or ‘interested’ in pursuing FP treatments after watching the video (same individual-transfemale—who was initially ‘very interested’ in pursuing FP treatments in the pre-video survey).

**Table 2.** Post-video survey results

Variable	Total population		AFAB		AMAB	
	n	Freq (%)	n	Freq (%)	n	Freq (%)
How would you rate your knowledge of FP after watching the video?	12		5		6	
Very good		4 (33.3)		2 (40.0)		2 (33.3)
Good		6 (50.0)		2 (40.0)		3 (50.0)
Fair		2 (16.7)		1 (20.0)		1 (16.7)
Not very good		0 (0.0)		0 (0.0)		0 (0.0)
Not at all		0 (0.0)		0 (0.0)		0 (0.0)
How would you describe your knowledge of FP after watching the video?	**12**		5		6	
I know and understand the specific details of FP		5 (42.7)		4		1 (16.7))
I know general principles but missing specific details		7 (58.3)		1 (20.0)		5 (83.3)
I have limited knowledge of FP		0 (0.0)		0 (0.0)		0 (0.0)
No knowledge of FP		0 (0.0)		0 (0.0)		0 (0.0)
How interested are you in knowing more about FP after watching the video?	**11**		5		5	
Very interested		0 (0.0)		0 (0.0)		0 (0.0)
Interested		2 (18.2)		1 (20.0)		1 (20.0)
Neutral		3 (27.3)		2 (40.0)		1 (20.0)
Not interested		3 (27.3)		1 (20.0)		1 (20.0)
Not at all		3 (27.3)		1 (20.0)		2 (0.4)
How comfortable are you discussing FP with a healthcare provider?	**11**		5		6	
Very comfortable		2 (18.2)		1 (20.0)		1 (16.7))
Comfortable		5 (45.4)		1 (20.0)		4 (66.7)
Neutral		3 (27.3)		2 (40.0)		1 (16.7))
Not Comfortable		1 (9.1)		1 (20.0)		0 (0.0)
Not at all		0 (0.0)		0 (0.0)		0 (0.0)
After watching the video, are you interested in pursuing FP treatment?	**11**		5		0	
Very interested		1 (9.1)		0 (0.0)		0 (0.0)
Interested		0 (0.0)		0 (0.0)		0 (0.0)
Neutral		1 (9.1)		1 (20.0)		0 (0.0)
Not interested		3 (27.3)		2 (40.0)		0 (0.0)
Not at all		6 (54.5)		2 (40.0)		0 (0.0)

### Video feedback

Twelve participants (12) completed the video feedback survey (completion rate of 57.1%) (Table 3). The overall feedback received was largely positive: 9/12 (81.8%) participants found the video to be useful, 10/11 (90.9%) were satisfied with the video, 11/12 (91.7%) understood the video and 12/12 (100.0%) reported FP to be an important topic. All participants (11/11; 100.0%) reported the language used and information provided in the video to be adequate, and 9/12 (75.0%) noted they would recommend the video to a friend in a similar situation to them. Nine participants (9) responded to an open-text question regarding what they felt was missing from the video: patients wanted to know more about the cost of FP (2/9; 22.2%) or wanted more specific details about the ‘procedural’ and specific aspects of FP (age and stage of puberty required to start FP, sperm collection process, the impact of pre-existing hormonal therapy on FP). One participant (1; 11.1%) praised the video, noting that it promoted understandability of FP and that it would help individuals to ‘determine if they are interested or not’. Constructive feedback received from eight participants included a desire for ‘reproductive system removal’ to be discussed (1/8; 12.5%) and modifying language from ‘assigned [sex] at birth’ to ‘a person whose body produces [gametes]’ (1/8; 12.5%). One participant also noted that they felt the messaging in the video implied that FP is ‘something [trans people] have to do’ (1/8; 12.5%). Positive feedback included that the video was ‘descriptive’ (1/8; 12.5%), ‘easy to understand' (1/8; 12.5%), and ‘did a good job of avoiding discussion of genitalia’ (1/8; 12.5%) that could lead to transfeminine individuals experiencing ‘dysphoria’ or ‘general [discomfort]’. Another participant also appreciated the ‘inclusive’ language used and noted the video did not induce feelings of discomfort, thereby supporting future ‘open dialogue’ with healthcare providers. They also reported the video ‘makes the decision [to pursue FP] very clear and information based’.

**Table 3.** Video survey feedback

Variable	Total population	
	n	Freq (%)
Did you find the video useful?	**11**	
Very useful		3 (27.3)
Useful		6 (54.5)
Neutral		2 (18.2)
Not very useful		0 (0.0)
Not at all		0 (0.0)
Are you satisfied overall with the video?	**11**	
Very satisfied		2 (18.2)
Satisfied		8 (72.7)
Neutral		1 (9.1)
Not very satisfied		0 (0.0)
Not at all		0 (0.0)
Did you understand the video?	**12**	
Completely		11 (91.7)
Most of it		0 (0.0)
Half of it		1 (8.3)
Less than half of it		0 (0.0)
Not at all		0 (0.0)
Did you feel this was an important topic to address?	**12**	
Very important		4 (33.3)
Important		8 (66.7)
Neutral		0 (0.0)
Not Important		0 (0.0)
I’m not sure		0 (0.0)
Did you find the language of the video adequate?	**11**	
Yes		11 (100.0)
No		0 (0.0)
I’m not sure		0 (0.0)
Did you find the information in the video adequate?	**11**	
Yes		11 (100.0)
No		0 (0.0)
I’m not sure		0 (0.0)
Would you recommend this video to a friend in a similar situation as you?	**12**	
Yes		9 (75.0)
No		0 (0.0)
I’m not sure		3 (25.0)

## DISCUSSION

FP preservation discussion and treatment is a key component of transgender care (5,6). Current medical literature on this issue, however, is scant, with minimal educational tools and resources for gender-diverse adolescents (7). Previous studies reviewing the attitudes, knowledge, and beliefs of transgender adolescents and young adults have demonstrated that knowledge of FP remains low (8–11). In a study by Riggs and Bartholomaeus (12), 68% of patients reported not knowing what their fertility options were prior to starting gender-affirming therapy. These results suggest an important knowledge gap in FP for gender-diverse youth, similar to the lack of knowledge of FP reported by our patient population (Table 1: pre-video survey results). In accordance with our initial hypothesis, the educational videos evaluated in this study increased overall knowledge of FP in transgender youth as shown by the statistically significant improvement in FP-related knowledge reported by study participants in the pre- and post-video surveys [FP knowledge in the post-video survey was rated as ‘very good’ or ‘good’ by ten participants (10/12; 83.3%), a statistically significant improvement from the pre-video survey (P = 0.039) in which only 6.7% of participants rated their knowledge of FP as ‘very good’ or ‘good’]. This suggests a positive impact of educational videos in the dissemination of knowledge of FP, with a specific focus on the unique needs of gender-diverse youths as presented here.

The improvement in perceived knowledge from participants after watching the videos did not, however, translate into an impact on the number of participants wanting to pursue FP with two participants showing interest in pursuing FP treatments in the pre-video survey and only one in the post-video survey. These results are concordant with previous reports on the use of FP therapy in gender-diverse adolescents with a reported 5% of patients opting for such treatment (13–15). This is also represented in our study with most adolescents not identifying having biological children as a goal for their future self, with adoption representing a solution for half the participants sampled. Those results are similar to previously published data, which demonstrated a significant desire to become a parent in the future in gender-diverse adolescents (66% of assigned females at birth and 67% of assigned males at birth sampled) with most respondents not envisioning having biological children (15). With FP being such an important pre-treatment consideration for gender-diverse youth and their families (12), this study demonstrates that whiteboard educational videos can be helpful and effective in supplementing FP discussions.

Comments from the open-ended questions in the post-video survey provided meaningful suggestions on what content would make the videos more inclusive and useful to patients and their families while providing helpful recommendations regarding the development of online educational tools for gender diverse youth. Overall satisfaction with the videos in this study suggested high patient satisfaction while language and information content were deemed adequate by participants. As described in previously published studies (16), whiteboard videos deliver concise, and reliable education in patient-friendly language with visuals. As such, broader dissemination of the FP videos produced by our team to other care centres and gender diversity clinics could positively impact the overall care of transgender youths.

Limitations of the study include its small sample size as well as the use of convenience sampling, which limits the generalization of its results and only permits observations about its findings. The study was also conducted in urban tertiary care centres with immediate access to FP specialists, which may not be representative of most centres providing care to gender-diverse youth. Furthermore, although results from the post-video survey revealed an overall increase in participant knowledge of FP, long-term information retention or impact on FP treatments cannot be inferred as the post-video assessment was done immediately after watching the video.
